# Inflexible Minds: Impaired Attention Switching in Recent-Onset Schizophrenia

**DOI:** 10.1371/journal.pone.0078062

**Published:** 2013-10-14

**Authors:** Henderikus G. O. M. Smid, Sander Martens, Marc R. de Witte, Richard Bruggeman

**Affiliations:** 1 University of Groningen, University Medical Center Groningen, University Center of Psychiatry, Groningen, The Netherlands; 2 University of Groningen, Neuroimaging Center, Groningen, The Netherlands; 3 University of Groningen, University Medical Center Groningen, Department of Neuroscience, Groningen, The Netherlands; Alexander Fleming Biomedical Sciences Research Center, Greece

## Abstract

Impairment of sustained attention is assumed to be a core cognitive abnormality in schizophrenia. However, this seems inconsistent with a recent hypothesis that in schizophrenia the implementation of selection (i.e., sustained attention) is intact but the control of selection (i.e., switching the focus of attention) is impaired. Mounting evidence supports this hypothesis, indicating that switching of attention is a bigger problem in schizophrenia than maintaining the focus of attention. To shed more light on this hypothesis, we tested whether schizophrenia patients are impaired relative to controls in sustaining attention, switching attention, or both. Fifteen patients with recent-onset schizophrenia and fifteen healthy volunteers, matched on age and intelligence, performed sustained attention and attention switching tasks, while performance and brain potential measures of selective attention were recorded. In the sustained attention task, patients did not differ from the controls on these measures. In the attention switching task, however, patients showed worse performance than the controls, and early selective attention related brain potentials were absent in the patients while clearly present in the controls. These findings support the hypothesis that schizophrenia is associated with an impairment of the mechanisms that control the direction of attention (attention switching), while the mechanisms that implement a direction of attention (sustained attention) are intact.

## Introduction

There is consensus that impairment of attention is a separable and core cognitive abnormality in schizophrenia [1]. The nature of this deficit is unclear, although it is very important to understand this deficit better for progress in pharmacological, neuroscience, and gene research [2,3]. One of the reasons for this state of affairs is the rather loosely-defined meanings in which the term ‘attention’ has been used in publications on attention in schizophrenia. A recent high-impact article stated on this subject that “… the term ‘attention’ can be defined so broadly that impaired performance on virtually any task could be construed as evidence for a deficit in attention” [4]. An abstract, but broadly accepted definition of attention is the ability to select a subset of the available information for preferential processing, while ignoring competing information. This applies, however, to many different mechanisms (e.g., space-based versus object-based attention), tied to many different levels of processing (e.g., selection at the level of sensory processing versus selection at the level of working memory). Deficits may exist at one level but not at another. For example, findings of impaired performance in schizophrenia patients on the classic Stroop color-word test of selective attention [5], are hard to allocate to a deficit at a specific level or in a specific mechanism [6–8]. Another example is that early Event-Related brain Potentials (ERPs) associated with selection in the visual modality [9–11] and the auditory modality [12,13] are diminished in schizophrenia patients when they perform sequential object-based selective attention tasks. When they perform spatial selection tasks, however, the ERPs suggest that early selective attention [14] and selection for visual working memory storage are intact [15]. 

In the present experiment we focus on hypotheses about the specific nature of attention impairments of schizophrenia patients. A long-held hypothesis is that patients have a sustained attention deficit [2,3,12,16] . This assumption is problematic, however. On the one hand, it is thought to be broadly supported by the many findings showing that schizophrenia patients are impaired in the performance of the Continuous Performance Task (CPT) [2,3,17]. On the other hand, the CPT variants most often found to be under-performed by patients [17] are actually attention switching tasks [18] (i.e., the CPTax [19] and the CPT-Identical Pairs [20]. Recently, it has been argued that schizophrenia is associated with an impairment of the control of information selection while the implementation of information selection is intact [4]. This hypothesis predicts that schizophrenia patients would be impaired in attention switching, but not in sustaining attention, because, as we argue below, attention switching, but not sustaining attention, engages mechanisms required to control the selection of information, while in both types of tasks a specified selection must be implemented.

In general, sustained selective attention is engaged in tasks in which attention is directed to the same stimulus on all stimulus presentations (i.e. on the basis of instructions), while selective attention switching is engaged in tasks in which attention is directed to different stimuli on different stimulus presentations (i.e. on the basis of precues and sometimes referred to as “transient attention” [18,21–24]). Sustaining and switching of attention can operate in the visual-spatial and the object domain of selection. For example, if during a number of sequential stimulus presentations attention has to be focused on always the same location in the visual field while ignoring other stimulated locations, the task is a sustained attention task in the visual-spatial domain. If between stimulus presentations the focus of attention has to be switched from one location to another (on the basis of cues), it is a spatial attention switching task. Sustained attention in the object domain is studied with tasks requiring attention to focus on one and the same stimulus (e.g., a letter or color) during sequential stimulus presentations. If the focus of attention has to switch from one stimulus (‘a’) to another (‘x’) between stimulus presentations, however, the task is an object-based attention switching task.

Although sustained attention and attention switching tasks usually differ in how long the same input must be attended, the crucial difference concerns the different mechanisms they engage. The issue, therefore, is not the *duration* of having to sustain attention. Sustained attention engages the mechanisms that enable the selection and preferential processing of one information source while ignoring competing information. Attention switching tasks engage these same mechanisms, but also call into play control mechanisms that enable the switching of the focus of attention, that is, mechanisms that enable flexibility in the selection of information. Spatial attention switching is made possible by control mechanisms that enable the focus of attention to disengage a currently focused location, move the focus to another location, and focus on that new location [25]. Attention switching, therefore, depends on mechanisms that control the direction of attention, which are not engaged during a state of sustained attention. Thus, sustained attention and attention switching tasks seem ideal to investigate, respectively, the implementation of selection and the control of selection, thought to be crucial for the investigation of attention deficits in schizophrenia [4].

Object-based attention studies in healthy volunteers show that in attention switching tasks target detection rates and selective attention related ERPs are smaller than in sustained attention tasks [18,22]. This indicates that having to switch selection diminishes early preferential target processing, resulting in target detection loss relative to maintaining a specific selection. Studies about switching selection in schizophrenia suggest there are deficits in the control of selection and not in the implementation of selection [4,26,27]. Here, we evaluate this hypothesis by testing its predictions that schizophrenia is associated with a deficit in attention switching (the control of selection), and not in sustained attention (the implementation of selection). To that end we compared the performance and ERP measures obtained from schizophrenia patients and healthy controls when they performed attention switching and sustained attention tasks. Because the CPT is one of the most often used tasks to study cognitive deficits in schizophrenia patients and will continue to be so in the near future [2,3], we applied two standard variants of this task [28] to implement sustained attention and attention switching conditions. 

These variants, the CPTx and CPTax [29] are very frequently applied in clinical settings and differ strongly in the need to switch attention to the target stimulus requiring an overt response [18,19]. In the CPTx, attention is oriented to an occasionally presented target letter (or digit) 'x' in a sequence of other non-target letters (or digits) ‘y’, presented at a rate of one per second. Only when this target is presented, the participant must make a response. During the entire task and on all stimulus presentations, attention is oriented in a sustained manner to the same target, and attention never needs to switch. Therefore, selection only needs to be implemented and not to be switched between different selections, representing a typical sustained attention task. In the CPTax, the instruction is to respond to the target ‘x’ only in an ‘a’ – ‘x’ stimulus sequence, but not in other sequences (‘y’ - ‘x’). The ‘a’ - ‘x’ sequence has a low probability (about .20) to be presented. In this case, attention is oriented to the letter ‘a’ but is summoned to switch to ‘x’ whenever ‘a’ is presented. In contrast to the CPTx, selection of the target ‘x’ for preferential processing in the CPTax is the result of a split-second switch of attention from ‘a’ to ‘x’. Correct target responses in the CPTax therefore depend on intact mechanisms that control changes in the focus of attention (i.e., on the control of selection).

In a previous study, we found that in healthy controls target detection rates were lower and selection related ERPs to targets smaller in the CPTax than in the CPTx [18], consistent with earlier findings with similar conditions [22]. In the present study we compared task performance and ERPs recorded with a new group of healthy volunteers, matched on age and general intelligence to a group of recent-onset schizophrenia patients.

There is a long history of ERP research on visual selective attention and its neural basis. This has produced several well established ERP measures of selective attention to sequentially presented stimuli [30–37] . These measures have often been applied to evaluate a wide variety of issues in fundamental research [38–44] and in clinical research [45–48]. Overall, these studies show that selection on the basis of a feature that stimuli have in common with a target-object, such as its color, (letter-) shape, or spatial frequency, leads to a very early modulation (about 150 ms after stimulus presentation) of the ERPs over the posterior scalp, usually called Selection Negativity (SN). Several studies have shown that deficits of selective attention are associated with a smaller amplitude of this potential [39,46,48]. One study found that the SN, while clearly present for healthy controls, was fully absent for schizophrenia patients in an attention switching condition [47].

On the basis of these previous findings we expected that, if schizophrenia is associated with a sustained attention deficit, they would produce more omission errors and smaller attention ERPs than controls to the target ‘x’ in the sustained attention CPTx. If schizophrenia is associated with an attention switching deficit, more omission errors and smaller attention ERPs are expected for patients than for controls to the targets in the CPTax. Thus, the hypothesis that schizophrenia is associated with a deficit in the control of selection, but not in the implementation of selection, would be supported if the patients’ performance and selection ERPs would not differ from the controls in the sustained attention task, but would be impaired in the attention switching task.

## Methods

### Participants

Fifteen in- and out-patients (10 males) were recruited from the University Center of Psychiatry at the University Medical Center Groningen and fifteen healthy controls (10 males) through advertisements. Inclusion criteria were an age between 18 and 40 years, being right-handed and, for the patients, a DSM-IV 295.xx diagnosis of schizophrenia established in the preceding 24 months. Exclusion criteria for all participants were a history of neurological disorders of the participant or a first relative, vision problems after correction, and drug dependence. An additional exclusion criterion for healthy participants was a history of psychiatric disorders of the participant or of a first relative. Starting at the time of admission to the University Center, the patients underwent an 8-week diagnostic protocol as part of standard-care procedures. Some of these patients were referred on the basis of acute psychosis while others were referred for re-assessment of their status in longitudinal care, ensured by the regular contact between clinicians and patients, and required by the mental health care system in the Netherlands for this group of patients. In this 8-week protocol, the data from clinical-diagnostic interviews, observations, heteroamnestic interviews and clinical records of the referring clinics and general practitioners were applied by SCAN trained senior psychiatrists to test in consensus the DSM-IV criteria for a DSM-IV 295.xx diagnosis of schizophrenia. The Positive and Negative Symptoms Scale (PANSS), obtained within a week distance of the week of testing, was used to assess the severity of current psychotic symptoms. Premorbid education level was scored on the basis of the highest level finished at the time of recruitment, with scores ranging from 1 (primary school) to 7 (university).

All fifteen patients had a DSM-IV 295.xx diagnosis of schizophrenia (paranoid n=10, disorganized n=1, schizophreniform n=1, schizoaffective n=2, undifferentiated n=1). They all used antipsychotics (risperidone n=7, olanzapine n=4, quetiapine n=1, clozapine n=3) with an averaged mean chlorpromazine (CPZ) equivalent [49] of 278.89 mg/d (SD 105.39). Demographic and clinical data are presented in Table 1.

**Table 1:** Group Means (SD) of Demographic, Clinical, Performance and ERP Data.

	Scores		P-values t-tests
	Controls	Patients	
Age (years)	26.4 (7.0)	24.3 (3.9)	ns
IQ	104.1 (13.4)	95.6 (13.4)	ns
Education	4.2 (1.3)	4.0 (1.4)	ns
PANSS Pos	N/A	10.9 (2.6)	N/A
PANSS Neg	N/A	14.8 (5.0)	N/A
PANSS Gen	N/A	28.6 (9.0)	N/A
Disorganization P2	N/A	1.87 (0.92)	N/A
CPZ eq dose/d	N/A	278.89 (105.39)	N/A
RT CPTx (ms)	513 (48)	555 (86)	ns
**RT CPTax (ms**	**435 (53)**	**503 (100)**	**0.028**
Omissions CPTx (perc)	0.4 (0.83)	4.27 (6.51)	ns
**Omissions CPTax (perc)**	**1.87 (2.39)**	**9.33 (14.49)**	**0.059**
False Alarms CPTx (perc)	0.27 (0.46)	0.53 (0.99)	ns
False Alarms CPTax (perc)	0.73 (1.16)	1.27 (1.75)	ns
SN targets CPTx (μV)	-4.1 (3.0)*	-3.3 (2.3)*	ns
**SN targets CPTax (μV)**	**-2.4 (2.5**)*	**-0.1 (1.4**)**^ns^**	**0.004**
SN cues CPTax (μV)	-2.2 (2.0)*	-1.6 (3.4)^ns^	ns
**SN unexpected targets CPTax (μV)**	**-2.8 (1.3**)*	**-0.8 (2.8**)**^ns^**	**0.023**

CPTx: sustained attention, CPTax: attention switching. Symbols indicate significance level of one-sample t-tests of the target minus non-target difference potentials: * denotes p < .002 and ns denotes no significant difference.

The study was approved by the Ethics Committee of the University Medical Center of Groningen (METc UMCG). The patients were asked whether they were interested in participating in the study. When they responded positively, they received a letter with information describing the purpose, in- and exclusion criteria, and procedures of the experiment. They were asked to carefully read this information and had the opportunity to ask any questions. After this, they had minimally one week time to decide on their participation. All patients were assessed for the capacity to consent by Dr. R. Bruggeman (MD, PhD) and Dr. H. Knegtering (MD, PhD). This was based on establishing their understanding of the purpose and procedures of the study and on establishing the absence of severe psychotic symptoms (see also Table 1, PANSS scores). Only those patients who by this procedure were found to be capable to consent were included in the study, so that it was not necessary to ask next of kin, care takers or guardians to provide consent on behalf of the patients. All participants provided written consent by themselves. Exclusion criteria were checked by interview.

Patients and controls did not differ significantly in education (t < 1, p = .686), intelligence (t = 1.748, p = .091) and age (t = 1.028, p = .313). Note in Table 1, that the PANSS scores indicate that the severity of psychosis was low or in remission at the time of testing (highest PANSS scores were for Positive items 16, for Negative items 26 and for General items 52). Cognitive disorganization as a symptom or symptom factor may be related to cognitive deficits in neuropsychological tasks [50], but in the present sample was too low and invariant to be used as a clinical correlate. The mean Conceptual Disorganization item-score of the positive symptom scale of the PANSS (P2) was 1.87, with 12 patients scoring 1 or 2, one scoring 3 and one scoring 4.

### Stimuli

 Three sets of letters (C,Q,G,D), (N,H,M,W), (I,L,J,T) were used as the stimuli. These letters are easy to discriminate between sets, but hard to discriminate within a set [41,51]. Two letters from one set served as target letters (‘ x1’ and ‘x2’) demanding a response. One letter from the second set was instructed as the cue (‘a’) for switching attention in the CPTax, while in the sustained attention CPTx it served as a non-target letter (‘y’). All letters from the third set served as non-targets (‘y’). The presentation probabilities were 0.17 for a target; 0.22 for the cue-letter; and 0.61 for the non-targets. These letters were presented in random order on a PC-controlled video monitor as white letters on a black background subtending 1.5° X 1.5° of visual angle.

### Procedure

 The participants were seated in a sound- and light-attenuated chamber at a table with a response panel and video monitor, with their index fingers resting on two response buttons. The task consisted of a randomized sequence of 164 stimulus presentations, repeated 6 times with sustained attention instructions (CPTx) and 6 times with attention switching instructions (CPTax). The order of the attention instructions and that of the blocks with different target letters was counterbalanced between participants. The stimuli were presented for 150 ms. The inter-stimulus interval, measured from one stimulus onset to the next, was 1300 ms.

The participants were first shown the relevant letters for the upcoming block for ten seconds (for example, ‘G’ and ‘Q’ in the CPTx, and ‘G’, ‘Q’ and ‘H’ in the CPTax). They were told that these letters would occasionally be presented randomly in a sequence of other letters. For sustained attention blocks (CPTx) they were told that the ‘G’ demanded a left-hand response and the ‘Q’ a right-hand response. They were further told that they had to respond as fast and accurately as possible to these letters. For the attention switching blocks (CPTax), they were told that only if cue ‘H’ was presented, the ‘G’ and ‘Q’ target letters demanded a fast and accurate response. Although these CPTs differed from standard versions because a response choice between two targets was required, it is a well-established fact that if response choice is inserted in two task conditions, it does not alter the performance and attention-related ERP differences between those conditions [18,43,52]. Further, the ERP and performance findings associated with attention switching in a no-choice detection task [22], have been replicated with a choice task [18]. Together, this justifies the argument that the present results can reliably be generalized to no-choice CPT versions. 

The participants were asked to make as little as possible eye movements and blinks. They performed short training blocks (of 1 minute) until performance had stabilized, before commencing the two experimental blocks with the instructed target letters. After each block, the participant received performance feedback on the video screen. After two experimental blocks with targets from the first letter set, the participants received new target-response assignments (with letters from the second letter set), practiced these and performed two experimental blocks. After this, they again received new target-response assignments with targets from the third letter set, practiced these and performed the final two experimental blocks. This was done for both tasks, so there were three pairs of blocks for each task, i.e., 12 blocks overall.

### Recording and analysis

 The EEG was recorded from the scalp using silver-chloride electrodes located at the scalp sites F7, Fz, F8, C3, Cz, C4, Pz, PO7 (halfway between O1 and the midpoint of a line between P3 and T5) and PO8 (halfway between O2 and the midpoint of a line between P4 and T6). These electrodes were referenced to the left ear lobe. The F7, Fz, F8, C3, Cz, C4, and Pz sites are defined by the International 10-20 system. Of these sites, the signals from C3, Cz, C4, Pz, PO7, and PO8 were used off-line for the current relevant analyses. The parieto-occipital PO7 and PO8 sites are located over cortical areas that are known to be involved in visual selective attention [37,53]. Eyeblinks and -movements were monitored with electrodes at both outer canthi of the eyes (horizontal electro-oculogram; EOG) and above and below the right eye (vertical EOG). 

 The EEG signals were filtered with a bandpass of 0.01-70 Hz (half-amplitude cutoffs). All signals were digitized at a rate of 512 Hz. Automated artifact rejection on the EEG signals was performed off-line to eliminate data epochs contaminated by EEG artifacts, excessive muscle activity (with a criterion of 100 µV) and amplifier saturation (about 10% of all trials). The influence of horizontal and vertical eyemovements (saccades and blinks) on the EEG recording was corrected using the Gratton and Coles technique [54].

 The signals were synchronized to the onset of the stimuli and were averaged, separately for each participant and stimulus type (in the CPTx: targets and distractors, in the CPTax: cues (a), expected targets (x|a), unexpected targets (x|y), unexpected distractors (y|a) and repeated distractors (y|y)), over epochs of 1400 ms, starting 100 ms before onset of the stimulus and ending 1300 ms post-stimulus, correcting for differences in the 100 ms pre-stimulus baseline. Next, these averages were used for statistical analyses (T-Tests, MANOVA). Each average was based on approximately 160 epochs. 

The SN consists of the earliest difference in EEG activity elicited by target and non-target stimuli over parieto-occipital (PO7, PO8) electrode sites. To obtain the SN, we subtracted the ERPs elicited by non-targets (y) from the ERPs elicited by targets (x) and cues (a). This is a standard subtraction procedure, yielding ERP activity reflecting the selective processing of relevant stimuli (x, a) relative to irrelevant stimuli (y). In the CPTax, these subtractions were done separately for the cue (cue ‘a’ minus non-target ‘y’), expected targets (cued target x|a minus cued non-target y|a) and unexpected targets (non-cued target x|y – non-cued non-target y|y). We next determined the peak amplitude of the difference potentials in the 150 – 300 ms interval after stimulus presentation. In our previous study [18], we found that cued targets in the CPTax are preceded by an ERP called ‘Contingent Negative Variation’ (CNV [55]), an ERP wave with negative amplitude, maximal at the Cz electrode position, which late part is correlated with reaction time [56]. Its amplitude was measured in the CPTax at the Cz electrode in the interval 1150 – 1250 ms after cue presentation. 

Statistical analyses on performance measures and the amplitudes of the ERPs were performed with GLM Repeated Measures MANOVA and t-tests, with the factors hemisphere, task, stimulus-type and group. Estimates of effect size of significant effects are given by η^2^.

## Results

### Performance measures

Table 1 shows that, as expected, omission rates were larger in the CPTax than in the CPTx, (F1,28 = 16.40, p<.001, η^2^=.37). Within group analyses showed that this effect was significant in both groups (controls: F1,14 = 9.08, p=.009, η^2^ = .39 ; patients: F1,14 = 10.85, p=.005, η^2^ = .44). The increase of omissions was significantly larger in the patients than in the controls (task by group interaction: F1,28 = 4.98, p=.034, η^2^ = .15). Overall, omission frequency was not significantly different between the groups (F1,28 = 3.49, p=.072), but independent samples t-tests showed that patients had more omissions than controls in the CPTax (t28 = -1.97, p=.059) and no significantly different omission rates in the CPTx (t28 = -1.68, p=.105). False alarm rates were significantly larger in the CPTax than in the CPTx (F1,28 = 4.40, p=.045, η^2^ = .14) but showed no interaction with or main effect of group (F1,28 < 1.5, p=.22). In summary, more errors were made in the attention switching CPTax than in the sustained attention CPTx. In the CPTx, patients did not differ from controls in omission rate, but relative to the CPTx, their omission rate increased more than that of the controls in the CPTax 

To assess differences in response speed, the RTs were analyzed. The RTs were faster in the CPTax than in the CPTx (F1,28 = 113.44, p<.001, η^2^ = .80). Within group analyses showed that this effect was significant in both groups (controls: F1,14 = 154.35, p<.001, η^2^ = .92; patients: F1,14 = 24.83, p<.001, η^2^ = .64). The increase in response speed in the CPTax was smaller in patients than in controls (task by group interaction: F1,28 = 4.40, p=.045, η^2^ = .14). Patients overall had longer RTs than controls (F1,28 = 4.18, p=.05, η^2^ = .13). Independent samples t-tests showed that patients had longer RTs than controls in the CPTax (t28 = -2.3, p=.028) and no significantly different RTs in the CPTx (t28 = -1.64, p=.113).

### Brain potential measures

**Figure 1:**
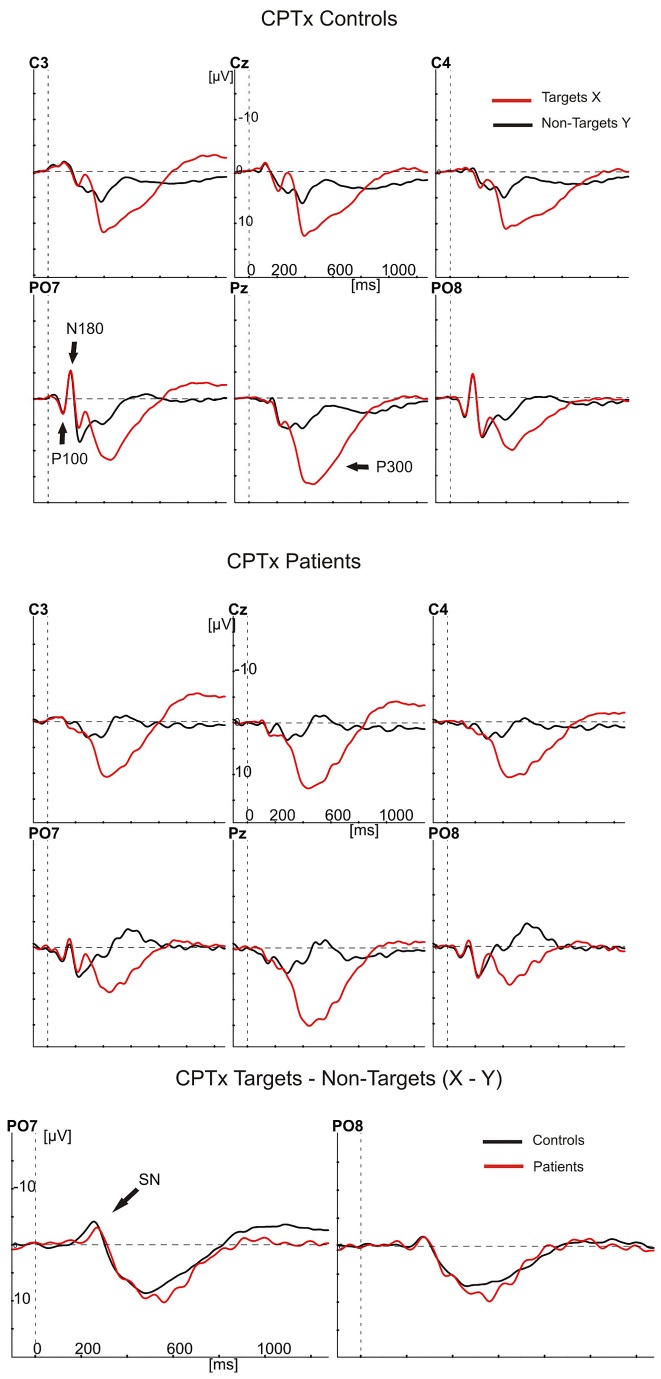
Grand averaged ERPs to targets (red) and non-targets (black) in the CPTx. The ERPs are shown of controls (upper 6 panels) and patients (middle 6 panels), at central (C3/z/4) and parieto-occipital electrode positions (PO7/8, Pz). Negative amplitudes are plotted upwards. At especially the PO7 electrode at about 200 ms, the red ERP (targets) has more negative amplitude than the black ERP (non-targets). This is the Selection Negativity (SN). The bottom two panels show the differences between the ERPs in the upper and middle panels as target minus non-target difference potentials at the PO7 and PO8 electrodes for patients (red) and controls (black).

**Figure 2:**
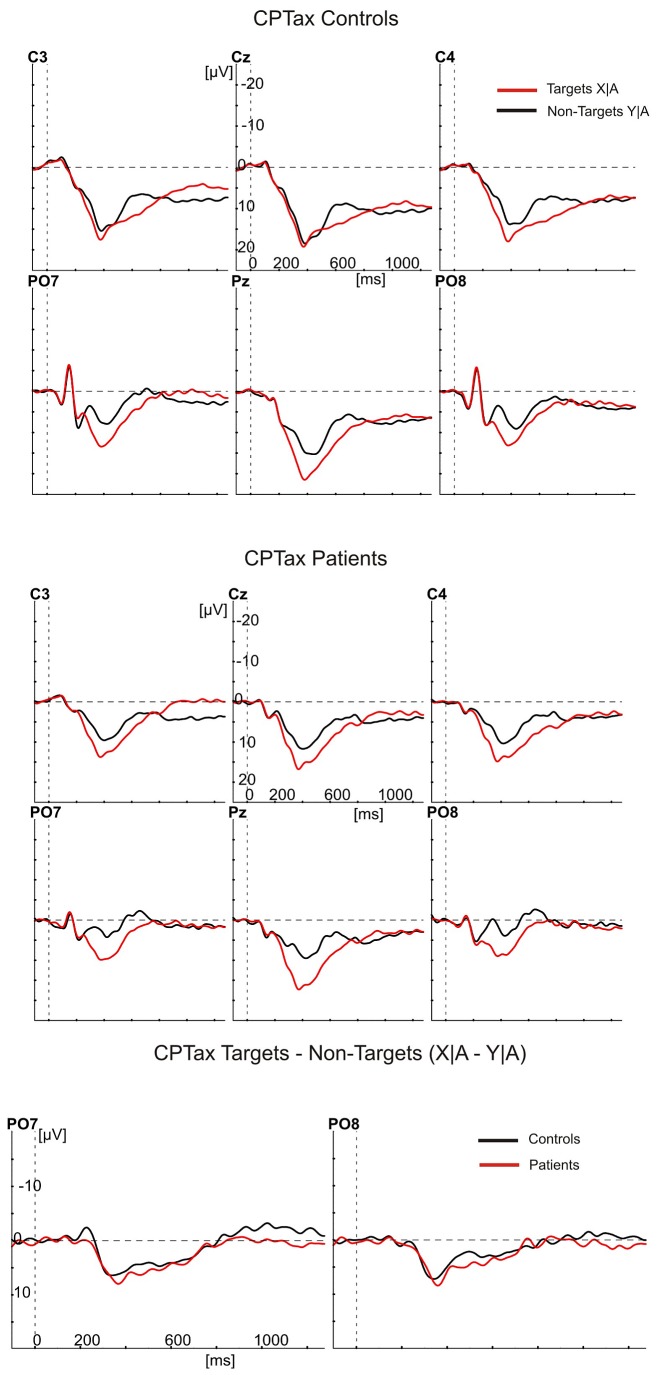
Grand averaged ERPs to cued targets (red) and cued non-targets (black) in the CPTax. The ERPs are shown of controls (upper 6 panels) and patients (middle 6 panels), at the same electrode positions as in Figure 1. The bottom two panels show the differences between the ERPs in the upper and middle panels as target minus non-target difference potentials at the PO7 and PO8 electrodes for patients (red) and controls (black).

**Figure 3:**
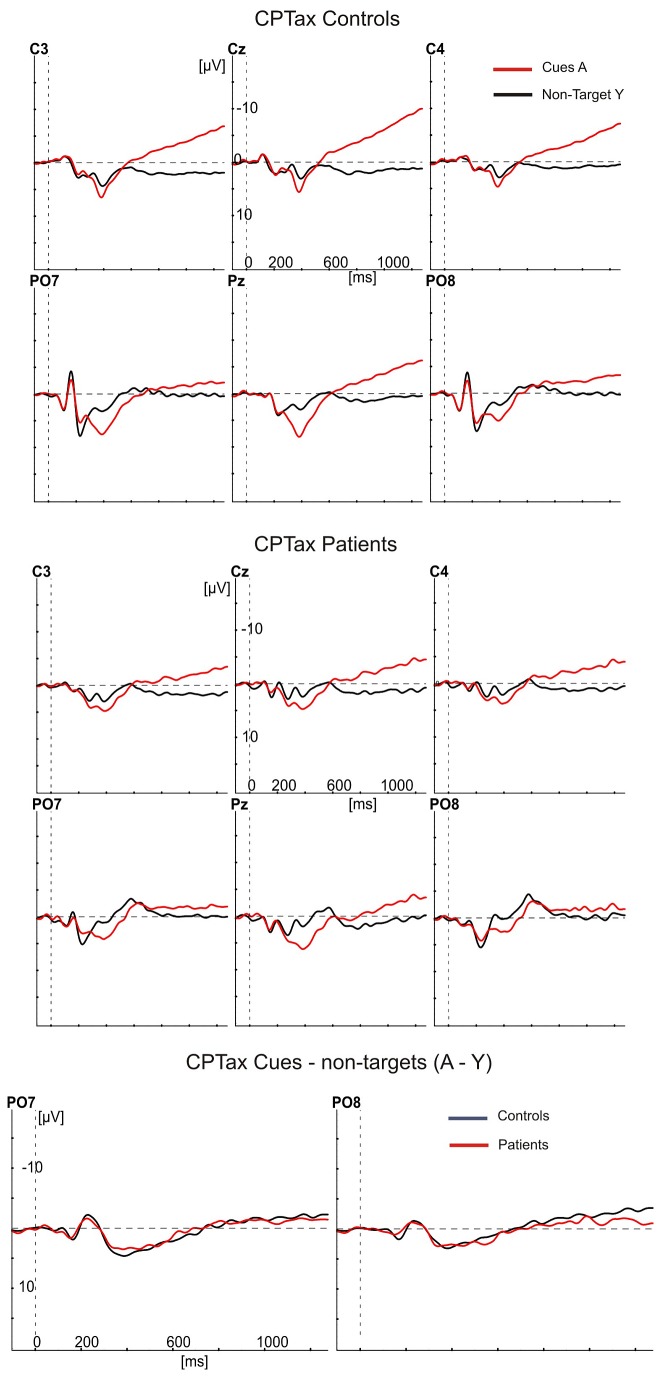
Grand averaged ERPs to cues (red) and non-cued non-targets (black) in the CPTax. The ERPs are shown of controls (upper 6 panels) and patients (middle 6 panels), at the same electrode positions as in Figure 1. The bottom two panels show the differences between the ERPs in the upper and middle panels as cue minus non-target difference potentials at the PO7 and PO8 electrodes for patients (red) and controls (black). The Contingent Negative Variation (CNV) is clearly visible at the Cz electrode as the large negative potential in the late part of the red waveform.

**Figure 4:**
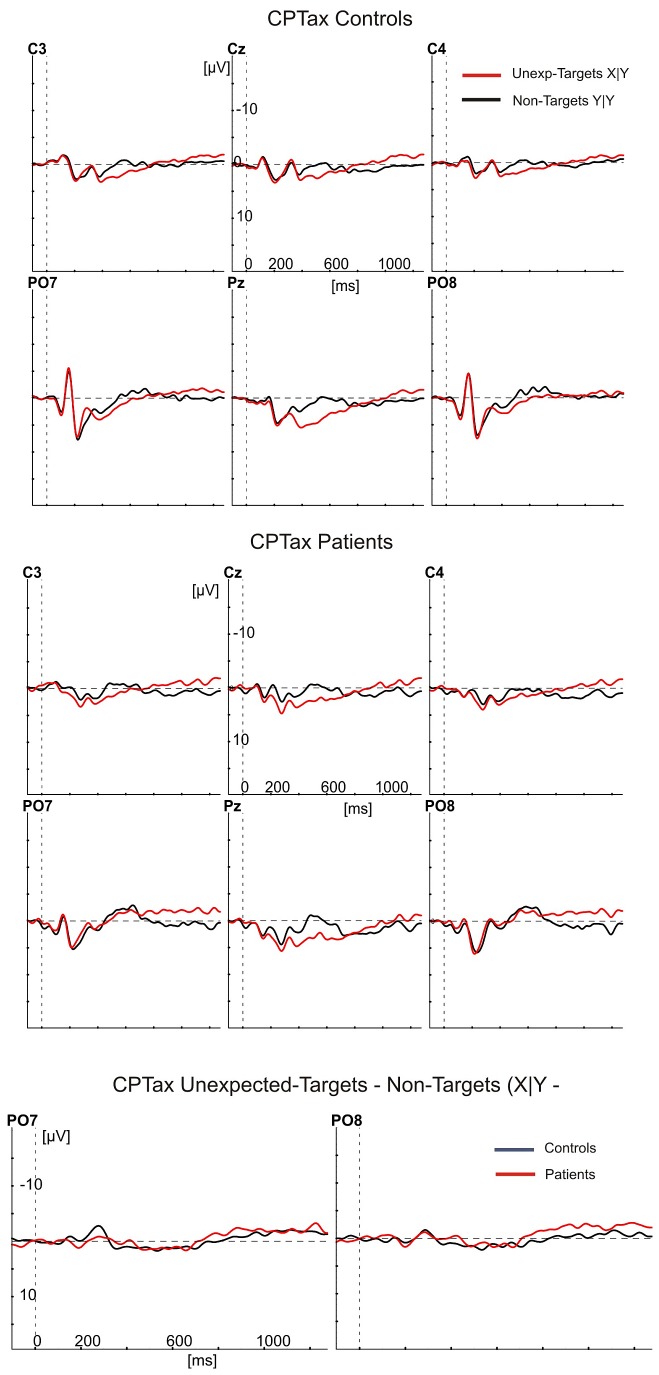
Grand averaged ERPs to unexpected targets (red) and non-cued non-targets (black) in the CPTax. The ERPs are shown of controls (upper 6 panels) and patients (middle 6 panels), at the same electrode positions as in Figure 1. The bottom two panels show the differences between the ERPs in the upper and middle panels as target minus non-target difference potentials at the PO7 and PO8 electrodes for patients (red) and controls (black).

Figures 1, 2, 3, and 4 show the ERPs (negative polarity upwards) elicited by targets and cues (red waveforms) and non-targets (black waveforms) at six electrode sites for controls (top panels) and patients (middle panels). The bottom panels show the differences between these ERPs as target minus non-target difference potentials for controls (black) and patients (red) at the PO7 and PO8 electrodes. In the top and middle panels, the ERPs to targets show a clear P300 component largest at the Pz electrode and peaking (downward) at about 400 ms after stimulus presentation. At the PO7 and PO8 electrodes, they also show the largest stimulus evoked potentials, consisting of the P100, N180 and P220. Note, that the earliest differences between target stimuli and non-targets started between the N180 and P220 at the PO7 electrode, with the red waveforms (targets) being more negative than the black waveforms. This difference concerns the Selection Negativity. 

An initial analysis was meant to replicate the finding in healthy controls that the SN is largest over the left hemisphere (PO7 [18,34,37,43]). If so, the ERPs at PO7 should be used to test the predictions about attention deficits. We therefore tested the effects of the factors hemisphere (left and right) and type of target stimulus (targets in the CPTx, in the CPTax the cues, targets preceded by the cue, and targets not preceded by the cue), on the peak amplitude of the target minus non-target difference potentials. As expected, the SN was largest over the left hemisphere (PO7: -2.17 µV [0.31]; PO8: -0.85 µV [0.24]; F1,28 = 28.47, p<.001, η^2^ = .50). In patients, however, these potentials were less lateralized (hemisphere by group interaction, F1,28 = 4.23, p=.049, η^2^ = .13). 

All further SN analyses were done with the amplitudes recorded at the PO7 electrode site. We first performed an overall analysis to test attention effects (sustained or switched), group effects and their interaction. The amplitude of the SN to the targets was smaller in the CPTax than in the sustained attention CPTx (F1,28 = 33.21, p<.001, η^2^ = .54). Within-group analyses showed that this effect was significant in both groups (controls: F1,14 = 6.97, p=.019, η^2^ = .33; patients: F1,14 = 33.87, p<.001, η^2^ = .71). Overall, the SN was smaller in patients than in controls (F1,28 = 4.36, p=.046, η^2^ = .14), but independent samples t-tests showed that patients had a smaller SN than controls in the CPTax (t28 = -3.14, p=.004) and no significantly different SN amplitude in the CPTx (t28 < 1, p=.33). The task by group interaction was not significant (F1,28 = 2.95, p=.097, η^2^ = .095). 

The latter, rather ambiguous result is probably due to a bottom-effect of the SN amplitude of the patients in the CPTax. As Figures 2 and 4 show, the expected and unexpected targets in the CPTax produced no component in the averaged ERPs of the patients that can be identified as the SN. To substantiate this observation, we performed two additional analyses. First we established whether a significant SN component was present for each task and group. This was done with one-sample t-tests on the difference between the ERP amplitude elicited by targets (or cues) and non-targets for each task and group. Table 1 shows the results of these tests. The SN was significant for all comparisons in the controls (all t14 < -3.8, all ps<.002), but in patients it was present only for the targets in the CPTx. Remarkably, for the patients no significant SN was present for any of the relevant stimuli of the CPTax. Patients had a significant SN to the targets in the CPTx (-3.3 µV, t14 = -5.6, p<.001), but in the CPTax, the ERPs to expected targets, unexpected targets and cues were not significantly different from the ERPs to the non-targets in the SN latency range (respectively, t14 < 1, p=0.75; t14 = -1.84, p=.09; t14 = -1.2, p=.27). 

Secondly, we determined separately for each group whether the amplitudes of the SN in the CPTx and CPTax were correlated. If no SN was present in the CPTax (leaving present only random variations in amplitude), no significant correlation between SN amplitude in CPTx and CPTax would exist. If an SN was present in the CPTax but a smaller version of it than in the CPTx, a significant correlation between the two should exist. For controls, the SN to targets in the CPTx was significantly correlated with the SN to all relevant stimuli in the CPTax (expected targets: p=.019; unexpected targets: p=.051; cues: p<.001). For the patients, however, it was not significantly correlated with the SN to expected (p=.125) and unexpected targets (p=.200) of the CPTax, while it was with the SN to the cues in the CPTax (p=.049). Together, these results indicate that controls had an SN to all relevant stimuli in both tasks, whereas in the ERP of the patients the SN to targets was present in the CPTx but absent in the CPTax. 

To evaluate the role of response preparation processes in (deficient) task performance, we analyzed the CNV. In the CPTax, the amplitude of the CNV at the Cz electrode in the interval 1150 – 1250 ms after cue presentation was significantly larger in response to a cue than to distractors during that same interval (F1,24 = 94.36, p<.001, η^2^ = .80). Patients had a smaller CNV (F1,24 = 6.89, p=0.015, η^2^ = .22) than controls (see Figure 3, Cz electrode).

## Discussion

This experiment evaluates the hypothesis that schizophrenia is associated with a deficit in switching the focus of attention but not with a deficit in sustaining the focus of attention. If confirmed, it would support the idea that in schizophrenia the control of selection is impaired, while implementation of selection is intact [4,26,27]. We tested the prediction that in a sustained attention task, task performance and stimulus selection ERPs of schizophrenia patients would not differ from healthy controls but would be diminished in an attention switching task. To that end, we asked patients with schizophrenia and matched healthy controls to perform two tasks, differing in whether target responses are based on sustained attention (CPTx), or are based on having to switch attention (CPTax). The requirement to switch the focus of attention engages mechanisms that control which information to select, in addition to the mechanisms that implement a selection in a sustained attention state [4,25].

The performance and ERP results from the healthy controls replicated those of previous studies [18,22]. In the attention switching task, omission rates were higher and SN amplitudes were smaller than in the sustained attention task. Thus, the requirement to switch attention rather than merely sustain attention resulted in less selective processing of the targets at a very early stage of processing (200 ms) and consequently in more missed targets. This underlines that attention switching requires more cognitive control than sustained attention, and suggests that having to control switching between selections, in addition to implementing them, consumes more limited resources than sustained implementation of a selection alone.

Our findings indicate that the patients with schizophrenia did not significantly differ from the controls in omission rate, RT, and amplitude of the SN in the sustained attention task. In the attention switching task, however, patients had slightly more omissions, significantly longer RTs, and significantly smaller SN amplitudes than the controls. Remarkably, none of the relevant stimuli in the CPTax that elicited a significant SN in the ERP of the controls did so in that of the patients. Finally, in the CPTax relative to the sustained attention CPTx, the patients had a significantly larger increase in omission rate and a significantly smaller increase in response speed than the controls. The decrease of the patients’ SN amplitude in the CPTax relative to that in the CPTx was also larger than that of the controls, but did not reach the .05 significance level, probably due to a bottom-effect resulting from the absence of a detectable SN for the patients in the CPTax.

These results, especially the absence of detectable SNs for patients in the CPTax, suggest that patients are impaired in switching attention from the cue to the target in the switching task, but not in maintaining attention to the targets in the sustained task. Together, they are inconsistent with the hypothesis that schizophrenia is associated with a sustained attention deficit [3]. In contrast, they do support the hypothesis that schizophrenia is associated with a deficit in the ability to switch attention, that is, with an impairment of the flexibility to adapt to varying task demands in the selection of information. The present finding that schizophrenia patients have no significant SN to relevant stimuli in the CPTax replicates earlier findings with a response precuing task [47]. These findings are also consistent with previous studies that presented performance indications of impaired covert attention switching in schizophrenia [57–60]. Taken together, the present findings further support the hypothesis that schizophrenia is associated with a deficit in the control of selection (attention switching), but not in the implementation of selection (sustained attention) [4].

Although the present pattern of results indicates that patients and controls did not significantly differ from each other in all measures of the CPTx, patients were somewhat slower, had slightly more omissions and false alarms, and had somewhat smaller SN amplitude than the controls (see Table 1). This could be taken as evidence that the significant differences between patients and controls present in the measures of the CPTax only represent an amplification of the small non-significant differences present in the CPTx. This argument, however, does not do justice to the fact that in the CPTax an additional mental process has to be performed relative to the CPTx, that is, a switch of attention from cue to target. This means that the CPTax does not engage the same, but harder to perform, mental operations as in the CPTx, and therefore is not just a more difficult version of the CPTx [see 18]. It seems more plausible that the small differences between the groups in the CPTx are due to other factors, for example increased fatigue [61] or a slightly lower level of arousal in the patients [62].

An interesting aspect of the present results is that the differences between patients and controls were more pronounced in the SN brain potential than in the behavioural measures. This has been reported also by other authors [62]. Since the SN is a measure of selective processing at the perceptual level [37], it may be that a mechanism at a higher level in the processing chain is able to (partially) compensate for the perceptual deficiency observed with the SN, leading in the end to near-normal behavioural output. One of our future research aims is to shed more light on this issue.

The performance and ERP results from the healthy controls also replicated another previous finding [18]. In the CPTax, RTs were faster and CNV amplitudes larger than in the CPTx. The CNV amplitude was significantly correlated with RT only. This indicates that in the CPTax there was more motor preparation in advance of the target than in the CPTx, leading to faster responses. Together with the selective attention ERPs, this supports the hypothesis that the CPTax engages two separable cognitive functions, switching of selective attention and transiently turning-on of motor preparation. The results showed that patients had a smaller CNV amplitude and a smaller increase in response speed. That is, patients not only had problems with switching attention from cue to target, but also with turning-on motor preparation. This may be explained by impaired selective processing of the cue [47], since the patients had no significant SN to the cue. Failure to turn on motor preparation may be the direct result of problems with switching attention from cue to target, but may also be an independent deficit, as suggested by other studies [63].

One may argue that the smaller SN amplitude of the patients to targets in the CPTax, was a secondary effect of diminished preparation, as indicated by the smaller target-preceding CNV amplitude in patients. However, the finding that patients also had a smaller SN than controls to unexpected targets, which were not preceded by preparatory activity, rules out this possibility. It could also be argued that the diminished SN to targets in the CPTax of the patients was influenced by their smaller P100 and N180 Evoked Potentials (e.g., Figure 1). This is unlikely because the Evoked Potentials of patients in the CPTx were also smaller than those of the controls, but the SN to targets was not significantly affected. Moreover, it is known for a long time that the P100 and N180 are so-called exogenous (evoked) potentials, whereas the SN is an endogenous potential that overlaps the Evoked Potentials [33].

One more result deserves discussion. This concerns the processing of the cues in the CPTax. One may wonder why the SN to the cues is visually present for patients (See Figure 3) and not significantly different between patients and controls (see Table 1), whereas the SN to expected and unexpected targets were visually not present and significantly different between patients and controls. Recall, that in the CPTax one first has to attend to the cue across a number of (nontarget) stimulus presentations, and only when the cue is presented, one has to switch attention to the target. Therefore, in the CPTax, attention to the cue has a more sustained nature than attention to the targets, albeit less than attention to the targets in the CPTx. The present finding that patients showed a visually detectable SN to the cues that was not significantly different from that of the controls clearly supports this analysis and further strengthens our interpretation that patients have no sustained attention deficit.

One potentially important alternative explanation of the present results should be considered. Clinical and experimental evidence have amply demonstrated that patients with schizophrenia have impairments in the perception of objects, consisting of loosened figure-to-ground organization and deteriorated perception of an object as an integrated whole [64–69]. These impairments are most probably due to a failure of automatic processes, because normal object perception is the result of pre-attentive (that is, automatic) mechanisms that operate according to Gestalt principles [64]. Since normal operation of higher cognitive functions (e.g., the control of information selection) depends on intact object perception, a deficit of object perception may lead to abnormal higher cognitive functioning. Therefore, the impairment of the switching of the focus of attention observed here with the patients, may not represent a primary deficit, but a secondary consequence of impaired object (i.e., letter) perception. In a recent experiment, we demonstrated that impaired object perception in schizophrenia indeed influences the switching of attention in the visual-spatial domain [70]. It is, therefore, not entirely clear whether failures of attention found in experiments with schizophrenia patients should be ascribed to a primary deficit of attention, or to a primary deficit of (pre-attentive) perception, leading to a secondary deficit of attention.

The present findings are consistent with earlier findings about impairments of schizophrenia patients in different CPT variants ([71]. They suggest that in schizophrenia the mechanisms that control the focus of selective attention have problems to change that focus on the basis of cues in a dynamic environment. In daily life this deficit could underlie observations of ‘cognitive slowness’, weak representations of the (social) environment [72], and impaired adaptation to changing daily task goals. Impairment of the control over which information should be selected for a current goal may also be related to the emergence of delusions, the experience of hallucinations, and negative symptoms, which are the primary symptoms of schizophrenia. 

Furthermore, control over the switching of the focus of attention is a critical component in the performance of many neuropsychological tests like the traditional Stroop test and the Trail making test. Therefore, if patients are impaired in attention switching the often reported under-performance of schizophrenia patients on these tests may be mainly determined by this impairment. Finally, the present findings were found in recent-onset (< 2 years) schizophrenia patients, and may not be generalizable to chronic patients, as has been found for spatial-attention switching impairments in schizophrenia [58]. It seems important that future studies address these issues.

We conclude that schizophrenia is associated with a deficit of switching the focus of attention, that is, of the control of information selection, but not with a deficit of maintaining selective attention, that is, of the implementation of selection. 

## References

[1] Nuechterlein KH, Barch DM, Gold JM, Goldberg TE, Green MF et al. (2004 ) Identification of separable cognitive factors in schizophrenia. Schizophr Res 72: 29-39. doi:10.1016/j.schres.2004.09.007. PubMed: 15531405. 15531405

[2] Gold JM, Thaker GK (2002) Cognitive phenotypes of schizophrenia: Attention. J Nerv Ment Dis 190: 638-639. doi:10.1097/00005053-200209000-00010. PubMed: 12357099. 12357099

[3] Nuechterlein KH, Green MF, Kern RS, Baade LE, Barch DM et al. (2008) The MATRICS Consensus Cognitive Battery, part 1: test selection, reliability, and validity. Am J Psychiatry 165: 203-213. doi:10.1176/appi.ajp.2007.07010042. PubMed: 18172019. 18172019

[4] Luck SJ, Gold JM (2008) The construct of attention in schizophrenia. Biol Psychiatry 64: 34-39. doi:10.1016/j.biopsych.2008.02.014. PubMed: 18374901. 18374901 PMC2562029

[5] Stroop JR (1935) Studies of interference in series verbal reactions. J Exp Psychol Hum Learn 18: 643–662.

[6] Chen EY, Wong AWS, Chen RYL, Au JW (2001) Stroop interference and facilitation effects in first-episode schizophrenic patients. Schizophr Res 48: 29–44. doi:10.1016/S0920-9964(00)00107-9. PubMed: 11278152. 11278152

[7] Henik A, Salo R (2004) Schizophrenia and the stroop effect. Behav Cogn Neurosci Rev 3: 42-59. doi:10.1177/1534582304263252. PubMed: 15191641. 15191641

[8] Kosmidis MH, Bozikas VP, Zafiri M, Karavatos A (2006) Shared cognitive processes underlying performance on the Wisconsin Card Sorting Test and the Stroop Test in patients with schizophrenia: A measurement artifact? Neurosci Lett 409: 234–238. doi:10.1016/j.neulet.2006.09.049. PubMed: 17030094. 17030094

[9] Alain C, Bernstein LJ, He Y, Cortese F, Zipursky RB (2002) Visual feature conjunction in patients with schizophrenia: an event-related brain potential study. Schizophr Res 57: 69-79. doi:10.1016/S0920-9964(01)00303-6. PubMed: 12165377. 12165377

[10] Potts GF, O’Donnell BF, Hirayasu Y, McCarley RW (2002) Disruption of neural systems of visual attention in schizophrenia. Arch Gen Psychiatry 59: 418- 424. doi:10.1001/archpsyc.59.5.418. PubMed: 11982445. 11982445

[11] Strandburg RJ, Marsh JT, Brown WS, Asarnow RF, Guthrie D et al. (1999) Continuous-Processing Related ERPS in Adult Schizophrenia: Continuity with Childhood Onset Schizophrenia. Biol Psychiatry 45: 1356–1369. doi:10.1016/S0006-3223(98)00349-7. PubMed: 10349042. 10349042

[12] Wood SM, Potts GF, Martin LE, Kothmann D, Hall JF et al. (2007) Disruption of auditory and visual attention in schizophrenia. Psychiatry Res 156: 105–116. doi:10.1016/j.pscychresns.2007.04.014. PubMed: 17889512. 17889512

[13] Alain C, Cortese F, Bernstein LJ, He Y, Zipursky RB (2001) Auditory feature conjunction in patients with schizophrenia. Schizophr Res 49: 179-191. doi:10.1016/S0920-9964(00)00138-9. PubMed: 11343876. 11343876

[14] Luck SJ, Fuller RL, Braun EL, Robinson B, Summerfelt A, et al. (2006) The speed of visual attention in schizophrenia: electrophysiological and behavioral evidence. Schizophr Res 85: 174-195. doi:10.1016/j.schres.2006.03.040. PubMed: 16713184. 16713184

[15] Gold JM, Fuller RL, Robinson BM, McMahon RP, Braun EL et al. (2006) Intact attentional control of working memory encoding in schizophrenia. J Abnorm Psychol 115: 658-673. doi:10.1037/0021-843X.115.4.658. PubMed: 17100524. 17100524

[16] Kurtz MM, Ragland JD, Bilker W, Gur RC, Gur RE (2001) Comparison of the continuous performance test with and without working memory demand in healthy controls and patients with schizophrenia. Schizophr Res 48: 307-316. doi:10.1016/S0920-9964(00)00060-8. PubMed: 11295383. 11295383

[17] Heinrichs RW (2001) In search of madness: Schizophrenia and Neuroscience. New York, NY: Oxford University Press.

[18] Smid HGOM, de Witte MR, Homminga I, van den Bosch RJ (2006) Sustained and transient attention in the Continuous Performance Task. J Clin Exp Neuropsychol 28: 859-883. doi:10.1080/13803390591001025. PubMed: 16822729. 16822729

[19] Servan-Schreiber D, Cohen JD, Steingard S (1996) Schizophrenic deficits in the processing of context. A test of a theoretical model. Arch Gen Psychiatry 53: 1105-1112. doi:10.1001/archpsyc.1996.01830120037008. PubMed: 8956676. 8956676

[20] Cornblatt BA, Risch NJ, Faris G, Friedman D, Erlenmeyer-Kimling L (1988) The Continuous Performance Test, identical pairs version (CPT-IP): I. New findings about sustained attention in normal families. Psychiatry Res 26: 223-238. doi:10.1016/0165-1781(88)90076-5. PubMed: 3237915. 3237915

[21] Eimer M (1996) ERP modulations indicate the selective processing of visual stimuli as a result of transient and sustained spatial attention. Psychophysiology 33: 13-21. doi:10.1111/j.1469-8986.1996.tb02104.x. PubMed: 8570791. 8570791

[22] Eimer M (1997) An Event-Related Potential (ERP) study of transient and sustained visual attention to color and form. Biol Psychol 44: 143–160. doi:10.1016/S0301-0511(96)05217-9. PubMed: 9043651. 9043651

[23] Heinze H-J, Mangun GR (1995) Electrophysiological signs of sustained and transient attention to spatial locations. Neuropsychologia 33: 889-908. doi:10.1016/0028-3932(95)00023-V. PubMed: 7477815. 7477815

[24] Mangun GR (1995) Neural mechanisms of visual selective attention. Psychophysiology 32: 4-18. doi:10.1111/j.1469-8986.1995.tb03400.x. PubMed: 7878167. 7878167

[25] Posner MI, Petersen SE (1990) The attention system of the human brain. Annu Rev Neurosci 13: 25–42. doi:10.1146/annurev.ne.13.030190.000325. PubMed: 2183676. 2183676

[26] Fuller RL, Luck SJ, Braun EL, Robinson BM, McMahon RP, et al. (2006) Impaired control of visual attention in schizophrenia. J Abnorm Psychol 115: 266-275. doi:10.1037/0021-843X.115.2.266. PubMed: 16737391. 16737391

[27] Wang K, Fan JT, Dong Y, Wang CQ, Lee TMC et al. (2005) Selective impairment of attentional networks of orienting and executive control in schizophrenia. Schizophr Res 78: 235-241. doi:10.1016/j.schres.2005.01.019. PubMed: 16154056. 16154056

[28] Gordon M (1986) Instruction manual for the Gordon diagnostic system. DeWitt, NY: Gordon Systems.

[29] Rosvold KE, Mirsky AF, Sarason I, Bransome ED, Beck LH (1956) A continuous performance test of brain damage. J Consult Psychol 20: 343-350. doi:10.1037/h0043220. PubMed: 13367264. 13367264

[30] Harter MR, Aine CJ (1984) Brain mechanisms of visual selective attention. In: Parasuraman R Davies DR Varieties of attention. Orlando, FL: Academic Press (pp. 293-321).

[31] Hillyard SA, Picton TW (1987) Electrophysiology of cognition. In: Plum F Handbook of Physiology, Sec 1. The nervous system: Vol. 5. Higher function of the nervous system, Part 2. (pp. 519-584). Baltimore, MD: American Physiological Society.

[32] Wijers AA, Mulder G, Okita T, Mulder LJ, Scheffers MK (1989) Attention to color: An analysis of selection, controlled search and motor activation, using event-related potentials. Psychophysiology 26: 89-109. doi:10.1111/j.1469-8986.1989.tb03137.x. PubMed: 2922460. 2922460

[33] Näätänen R (1992) Attention and brain function. Hillsdale, NJ: Erlbaum.

[34] Kenemans JL, Kok A, Smulders FTY (1993) Event-related potentials to conjunctions of spatial frequency and orientation as a function of stimulus parameters and response requirements. Electroencephalogr Clin Neurophysiol 88: 51-63. doi:10.1016/0168-5597(93)90028-N. PubMed: 7681391. 7681391

[35] Mangun GR, Hillyard SA (1995) Mechanisms and models of selective attention. In: Rugg MD Coles MGH Electrophysiology of mind: event-related potentials and cognition. New York, NY: Oxford University Press (pp. 86-131).

[36] Hillyard SA, Anllo-Vento L (1998) Event-related brain potentials in the study of visual selective attention. Proc Natl Acad Sci U S A 95: 781–787. doi:10.1073/pnas.95.3.781. PubMed: 9448241. 9448241 PMC33798

[37] Smid HGOM, Jakob A, Heinze H-J (1999) An event-related brain potential study of visual selective attention to conjunctions of color and shape. Psychophysiology 36: 264-279. doi:10.1017/S0048577299971135. PubMed: 10194973. 10194973

[38] Hillyard SA, Münte TF (1984) Selective attention to color and location: An analysis with event related brain potentials. Percept Psychophys 36: 185-198. doi:10.3758/BF03202679. PubMed: 6514528. 6514528

[39] Martens S, Munneke J, Smid HGOM, Johnson A (2006) Quick minds don't blink: electrophysiological correlates of individual differences in attentional selection. J Cogn Neurosci 18: 1423-1438. doi:10.1162/jocn.2006.18.9.1423. PubMed: 16989545. 16989545

[40] Molholm S, Ritter W, Javitt DC, Foxe JJ (2004) Multisensory visual-auditory object recognition in humans: a high-density electrical mapping study. Cereb Cortex 14: 452-465. doi:10.1093/cercor/bhh007. PubMed: 15028649. 15028649

[41] Rotte M, Heinze H-J, Smid HGOM (1997) Selective attention to conjunctions of color and shape of alphanumeric versus non-alphanumeric stimuli: a comparative electrophysiological study. Biol Psychol 46: 199-221. doi:10.1016/S0301-0511(97)00018-5. PubMed: 9360773. 9360773

[42] Smid HGOM, Boecker KBE, van Touw DA, Mulder G, Brunia CH (1996) A psychophysiological investigation of the selection and use of partial stimulus information in response choice. J Exp Psychol Hum Percept Perform 22: 3-24. doi:10.1037/0096-1523.22.1.3. PubMed: 8742249. 8742249

[43] Smid HGOM, Fiedler R, Heinze H-J (2000) An electrophysiological study of the insertion of response choice. J Exp Psychol Hum Percept Perform 26: 1053-1071. doi:10.1037/0096-1523.26.3.1053. PubMed: 10884009. 10884009

[44] Wills AJ, Lavric A, Croft GS, Hodgson TL (2007) Predictive learning, prediction errors, and attention: evidence from event-related potentials and eye tracking. J Cogn Neurosci 19: 843-854. doi:10.1162/jocn.2007.19.5.843. PubMed: 17488208. 17488208

[45] Baas JM, Kenemans JL, Mangun GR (2002) Selective attention to spatial frequency: an ERP and source localization analysis. Clin Neurophysiol 113: 1840-1854. doi:10.1016/S1388-2457(02)00269-9. PubMed: 12417240. 12417240

[46] Smid HGOM, Trümper BG, Pottag G, Wagner K, Lobmann R et al. (1997) Differentiation of hypoglycemia induced cognitive impairments: An electrophysiological approach. Brain 120: 1041-1056. doi:10.1093/brain/120.6.1041. PubMed: 9217687. 9217687

[47] Smid HGOM, Westenbroek JM, Bruggeman R, Knegtering H, van den Bosch RJ (2009) Abnormal externally-guided movement preparation in recent-onset schizophrenia is associated with impaired selective attention to external input. Psychiatry Res 170: 75-81. doi:10.1016/j.psychres.2008.06.007. PubMed: 19762086. 19762086

[48] van Laar MW, Volkerts ER, Verbaten MN, Trooster S, van Megen HJ et al. (2002) Differential effects of amitriptyline, nefazodone and paroxetine on performance and brain indices of visual selective attention and working memory. Psychopharmacology (Berl) 162: 351-363. doi:10.1007/s00213-002-1116-0. PubMed: 12172688. 12172688

[49] Andreasen NC, Pressler M, Nopoulos P, Miller D, Ho BC (2010) Antipsychotic dose equivalents and dose-years: a standardized method for comparing exposure to different drugs. Biol Psychiatry 67: 255-262. doi:10.1016/j.biopsych.2009.08.040. PubMed: 19897178. 19897178 PMC3677042

[50] Mass R, Schoemig T, Hitschfeld K, Wall E, Haasen C (2000) Psychopathological Syndromes of Schizophrenia: Evaluation of the Dimensional Structure of the Positive and Negative Syndrome Scale. Schizophr Bull 26: 167-177. doi:10.1093/oxfordjournals.schbul.a033437. PubMed: 10755679. 10755679

[51] Smid HGOM, Lamain W, Hogeboom MM, Mulder LJM, Mulder G (1991) Psychophysiological evidence for continuous information transmission between visual search and response processes. J Exp Psychol Hum Percept Perform 17: 696-714. doi:10.1037/0096-1523.17.3.696. PubMed: 1834785. 1834785

[52] Hackley SA, Schäffer R, Miller J (1990) Preparation for Donders' type B and C reaction tasks. Acta Psychol 74: 15-33. doi:10.1016/0001-6918(90)90032-B. PubMed: 2392955. 2392955

[53] Heinze HJ, Mangun GR, Burchert W, Hinrichs H, Scholz M et al. (1994) Combined spatial and temporal imaging of brain activity during visual selective attention in humans. Nature 372: 543-546. doi:10.1038/372543a0. PubMed: 7990926. 7990926

[54] Gratton G, Coles MG, Donchin E (1983) A new method for off-line removal of ocular artifact. Electroencephalogr Clin Neurophysiol 55: 468-484. doi:10.1016/0013-4694(83)90135-9. PubMed: 6187540. 6187540

[55] Walter WG, Cooper R, Aldridge VJ, McCallum WC, Winter AL (1964) Contingent Negative Variation: an electrical sign of sensorimotor association and expectancy in the human brain. Nature 203: 380-384. doi:10.1038/203380a0. PubMed: 14197376. 14197376

[56] Leuthold H, Sommer W, Ulrich R (1996) Partial advance information and response preparation: inferences from the lateralized readiness potential. J Exp Psychol Gen 125: 307-323. doi:10.1037/0096-3445.125.3.307. PubMed: 8830109. 8830109

[57] Early TS, Posner MI, Reiman EM, Raichle ME (1989) Hyperactivity of the left striato-pallidal projection. Part 1: Lower level theory. Psychiatr Dev 7: 85-108. PubMed: 2695925. 2695925

[58] Maruff P, Hay D, Malone V, Currie J (1995) Asymmetries in the covert orienting of visual spatial attention in schizophrenia. Neuropsychologia 33: 1205-1223. doi:10.1016/0028-3932(95)00037-4. PubMed: 8552225. 8552225

[59] Posner MI, Early TS, Reiman E, Pardo PJ, Dhawan M (1988) Asymmetries in hemispheric control of attention in schizophrenia. Arch Gen Psychiatry 45: 814-821. doi:10.1001/archpsyc.1988.01800330038004. PubMed: 3415424. 3415424

[60] Smith GL, Large MM, Kavanagh DJ, Karayanidis F, Barrett NA et al. (1998) Further evidence for a deficit in switching attention in schizophrenia. J Abnorm Psychol 107: 390-398. doi:10.1037/0021-843X.107.3.390. PubMed: 9715574. 9715574

[61] Gorissen M, Sanz JC, Schmand B (2005) Effort and cognition in schizophrenia patients. Schizophr Res 78: 199-208. doi:10.1016/j.schres.2005.02.016. PubMed: 16154055. 16154055

[62] Verleger R, Talamo S, Simmer J, Śmigasiewicz K, Lencer R (2013) Neurophysiological sensitivity to attentional overload in patients with psychotic disorders. Clin Neurophysiol 124: 881-892. doi:10.1016/j.clinph.2012.11.003. PubMed: 23357693. 23357693

[63] Verleger R, Wascher E, Arolt V, Daase C, Strohm A et al. (1999) Slow EEG potentials (contingent negative variation and post-imperative negative variation) in schizophrenia: their association to the present state and to Parkinsonian medication effects. Clin Neurophysiol 110: 1175-1192. doi:10.1016/S1388-2457(99)00023-1. PubMed: 10423184. 10423184

[64] Uhlhaas PJ, Mishara AL (2007) Perceptual anomalies in schizophrenia: integrating phenomenology and cognitive neuroscience. Schizophr Bull 33: 142-156. PubMed: 17118973. 17118973 10.1093/schbul/sbl047 PMC2632288

[65] Uhlhaas PJ, Silverstein SM (2005) Perceptual organization in schizophrenia spectrum disorders: empirical research and theoretical implications. Psychol Bull 131: 618–632. doi:10.1037/0033-2909.131.4.618. PubMed: 16060805. 16060805

[66] Silverstein SM, Keane BP (2011) Perceptual organization impairment in schizophrenia and associated brain mechanisms: Review of research from 2005 to 2010. Schizophr Bull 37: 690-699. doi:10.1093/schbul/sbr052. PubMed: 21700589. 21700589 PMC3122298

[67] Place EJ, Gilmore GC (1980) Perceptual organization in schizophrenia. J Abnorm Psychol 89: 409–418. doi:10.1037/0021-843X.89.3.409. PubMed: 7410708. 7410708

[68] Doniger GM, Foxe JJ, Murray MM, Higgins BA, Javitt DC (2002) Impaired visual object recognition and dorsal/ventral stream interaction in schizophrenia. Arch Gen Psychiatry 59: 1011-1020. doi:10.1001/archpsyc.59.11.1011. PubMed: 12418934. 12418934

[69] Sehatpour P, Dias EC, Butler PD, Revheim N, Guilfoyle DN et al. (2010) Impaired visual object processing across an occipital-frontal-hippocampal brain network in schizophrenia: an integrated neuroimaging study. Arch Gen Psychiatry 67: 772–782. doi:10.1001/archgenpsychiatry.2010.85. PubMed: 20679585. 20679585 PMC4283949

[70] Smid HGOM, Bruggeman R, Martens S (2013) Fragmented perception: Slower space-based but faster object-based attention in recent-onset psychosis with and without schizophrenia. PLOS ONE 8(3): e59983. doi:10.1371/journal.pone.0059983. PubMed: 23536901. 23536901 PMC3607576

[71] Elvevåg B, Weinberger DR, Suter JC, Goldberg TE (2000) Continuous performance test and schizophrenia: a test of stimulus-response compatibility, working memory, response readiness, or none of the above? Am J Psychiatry 157: 772-780. doi:10.1176/appi.ajp.157.5.772. PubMed: 10784471. 10784471

[72] Feinberg TE, Rifkin A, Schaffer C, Walker E (1986) Facial discrimination and emotional recognition in schizophrenia and affective disorders. Arch Gen Psychiatry 43: 276-279. doi:10.1001/archpsyc.1986.01800030094010. PubMed: 3954548. 3954548

